# On Diseases of the Spine and of the Nerves

**Published:** 1871-03

**Authors:** 


On Diseases of the Spine and of the Nerves. By Charles B. Radcliffe, M.D., F.R.C.P., London; John N. Radcliffe, J. W. Begbie, M.D., F.R.C.P., Edinburgh; Francis E. Ainstee, M.D., F.R.C.P. and Jno. R. Reynolds, M.D., F.R.S. Henry C. Lea, Publisher, Philadelphia; for sale by Cobb, Prichard & Co., 81–3 Lake Street, Chicago.

This is a small volume of 196 pages, comprising a series of essays extracted from the "System of Medicine," edited by J. Russell Reynolds, M.D., on a group of diseases of great interest, and many of them of frequent occurrence.

The subjects treated of comprise Meningitis, Myelitis, Spinal Congestion, Tetanus, Spinal irritation, General Spinal Paralysis, Hysterical Paraplegia, Reflex Paraplegia, Infantile Paralysis, Spinal Hemorrhage, Induration of the Spinal Cord, Atrophy and Hypertrophy of the Spinal Cord, Tumor of the Spinal Cord, Concussion of the Spine, Compression of the Cord, Caries of the Vertebral Column, Spina Bifida, Epidemic Cerebro-Spinal Meningitis, Neuritis and Neuroma, Neuralgia, Local Paralysis from Nerve Disease, Local Spasms, Torticollis, and Local Anæsthesia. The book presents the latest advances in the knowlege of these several subjects.

